# Ureterocolic Fistula Secondary to Diverticulitis of the Sigmoid Colon after Laparoscopic Salpingo-Oophorectomy: A Case Report and Literature Review

**DOI:** 10.1155/2019/6180534

**Published:** 2019-12-11

**Authors:** Hirohumi Yamanaka, Satoko Takeuchi, Nao Kirigaya, Akihito Kato, Satoshi Kaseki, Chika Sugita, Shigeko Saito, Tomomitsu Okamoto

**Affiliations:** Department of Obstetrics and Gynecology, Chukyo Hospital, 1-1-10, Sanjo, Minami-ku, Nagoya 457-8510, Japan

## Abstract

Ureterocolic fistula is a rare phenomenon and cases secondary to diverticulitis are even rarer. We present a case of ureterocolic fistula secondary to diverticulitis of the sigmoid colon following laparoscopic salpingo-oophorectomy due to endometriomas. To our knowledge, this is the first case that occurred in a patient with gynecologic surgery.

## 1. Introduction

Ureteral and vesical injury is one of the major complications related to surgical and gynecologic procedures such as hysterectomy and oophorectomy. Injuries are sometimes identified intraoperatively, but diagnosis of ureteral injury is often delayed. Moreover, ureterocolic fistula is extremely rare. The purpose of this report is to present a case of ureterocolic fistula in a 62-year-old-woman diagnosed more than three months following laparoscopic salpingo-oophorectomy due to endometriomas. A possible scenario for the development of the fistula is also discussed.

## 2. Case Report

A 62-year-old-woman, gravida 2, para 2, menopaused at 50 years old, was referred to our hospital for the operation of a pelvic tumor. Her past or familial history was unremarkable. CT scan of the abdomen and pelvis demonstrated a right adnexal mass suspicious of an endometrioma, measuring 6 cm in size. Preoperative laboratory data were all within normal limits including a CA125 level of 3.5 IU/ml (normal <35). The patient refused to have hysterectomy, and a laparoscopic bilateral salpingo-oophorectomy was performed. There were dense adhesions of cul de sac peritoneum. A probable endometrioma of the right ovary was adherent to the pelvic sidewall and to the posterior broad ligament. The normal-sized left ovary and tube were also adherent to the pelvic sidewall and to the uterus. First, left salpingo-oophorectomy was performed without difficulty utilizing the Harmonic scalpel (HS). Next, the right infundibulopelvic ligament was coagulated and divided using the HS. The dense adhesion of the right ovary and tube to the pelvic sidewall and uterus as well as the cul de sac adhesion to the posterior lower uterine segment were then lysed mostly by blunt detaching with occasional bipolar coagulation. Finally, the right ovary and tube were removed with the HS by coagulating and dividing its remaining attachments to the uterus. Two sheets of Interceed^TM^ were applied to the adhesiolysed area of the right pelvic sidewall and posterior broad ligament. The pathologic report confirmed a small endometrioma in the left ovary in addition to the one in the right ovary. The postoperative course was uneventful, and the patient was discharged on postoperative day 3.

**Figure 1:**
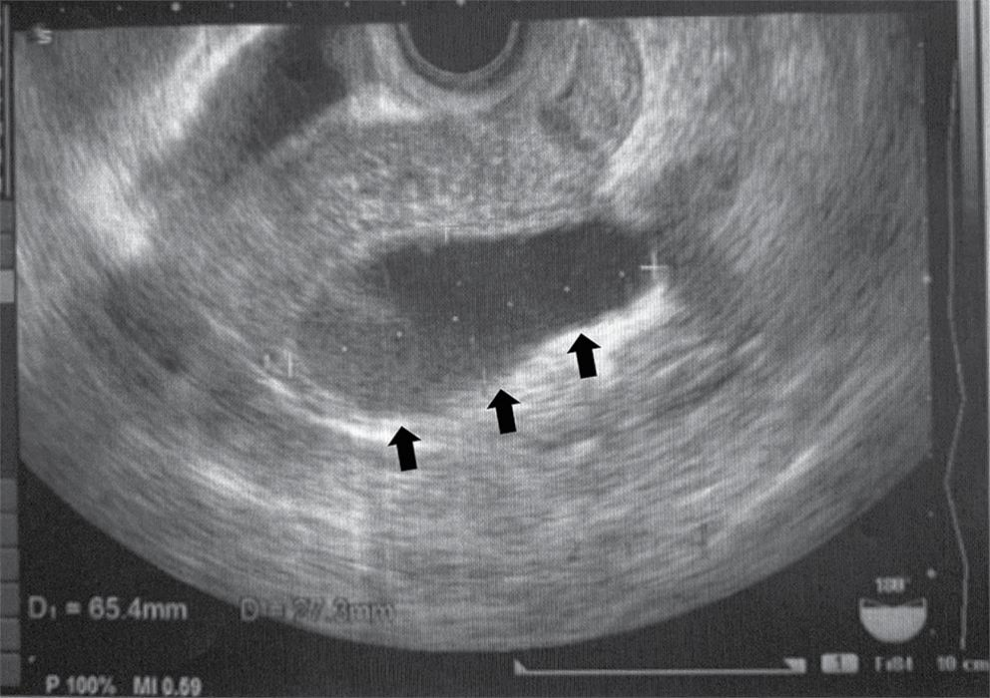
Transvaginal ultrasound on postoperative day 34 showing a hypoechoic cystic area adjacent to the uterus right posteriorly, measuring 65.4 mm × 27.3 mm.

The patient presented on postoperative day 11 complaining of right lower abdominal pain, which resolved without medication two days thereafter. Her next visit was on postoperative day 34 as the routine postoperative checkup. She was free of complaints, but transvaginal ultrasound showed a hypoechoic cystic shadow adjacent right posteriorly to the uterus (Figure 1).

**Figure 2:**
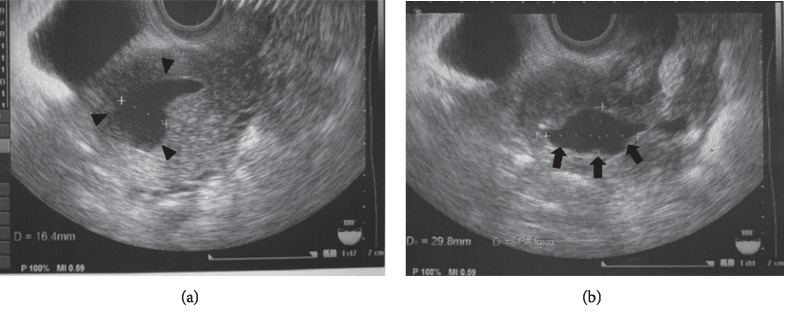
Transvaginal ultrasound on postoperative day 62 showing dilated uterine cavity ((a), arrowheads). The cystic echo observed on day 34 still exists but decreased in size ((b), arrows, 29.8 mm × 15.1 mm).

No ascites was observed. On postoperative day 62 she presented with a complaint of having thin vaginal discharge several times a day. She had no fever, and blood test was not performed. Transvaginal ultrasound revealed dilated uterine cavity suggestive of fluid retention (Figure 2(a)). The cystic hypoechoic shadow on day 34 still existed but decreased in size (Figure 2(b)).

**Figure 3:**
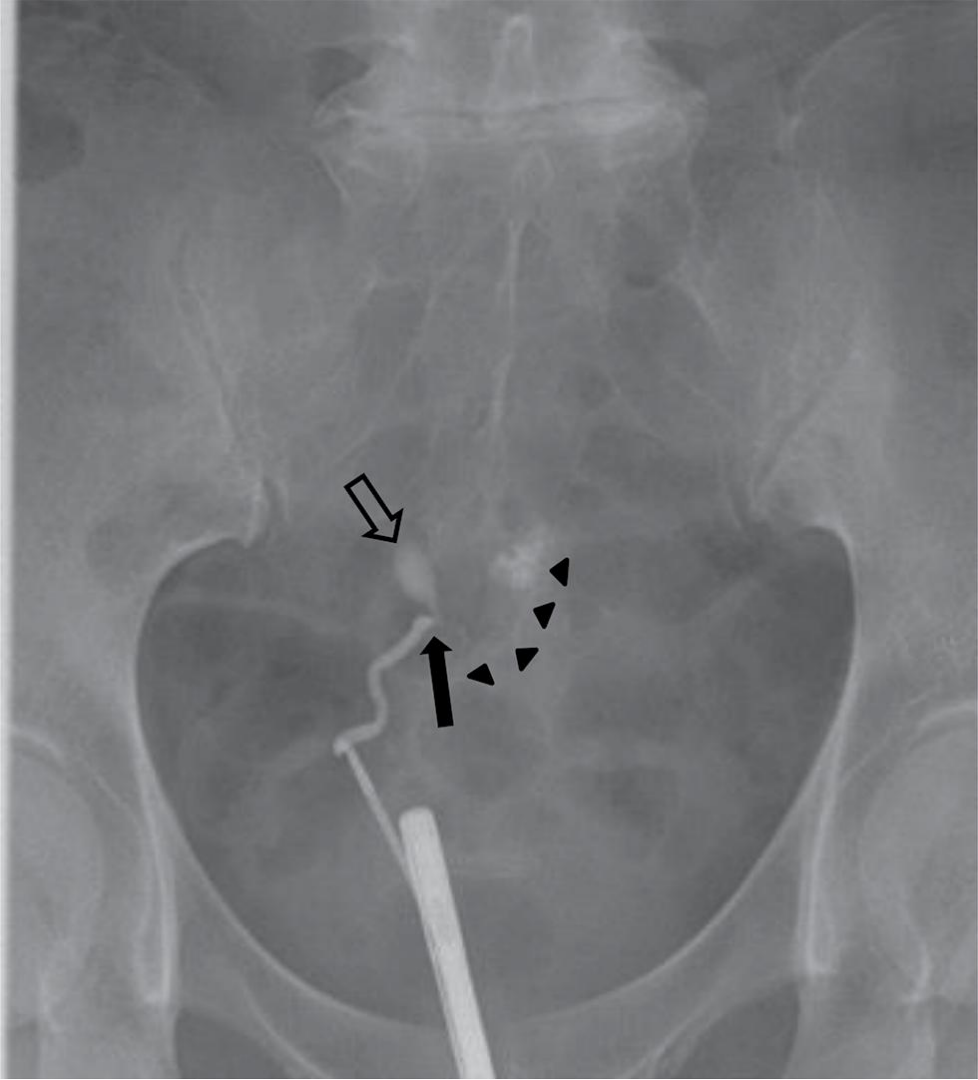
Right retrograde pyelography demonstrating stricture of the right ureter (arrow) and its deviation to the left side, from which a fistula into the sigmoid colon is observed (arrowheads). Extravasation of contrast into the mid-pelvis is also present right above the narrowing of the ureter (hollow arrow), suggestive of a fistula to the peritoneum.

The vaginal discharge gradually decreased and completely stopped around postoperative day 110. On the other hand, however, watery diarrhea, appetite loss and right flank pain appeared and persisted. On postoperative day 137 pelvic ultrasound showed hydronephrosis of the right kidney, and the patient was consulted to the urology department of our institution. Retrograde pyelography revealed that the right ureter was narrowed and shifted to the left at the level of the uterosacral ligament, with a fistula leading to the sigmoid colon (Figure 3). 

**Figure 4:**
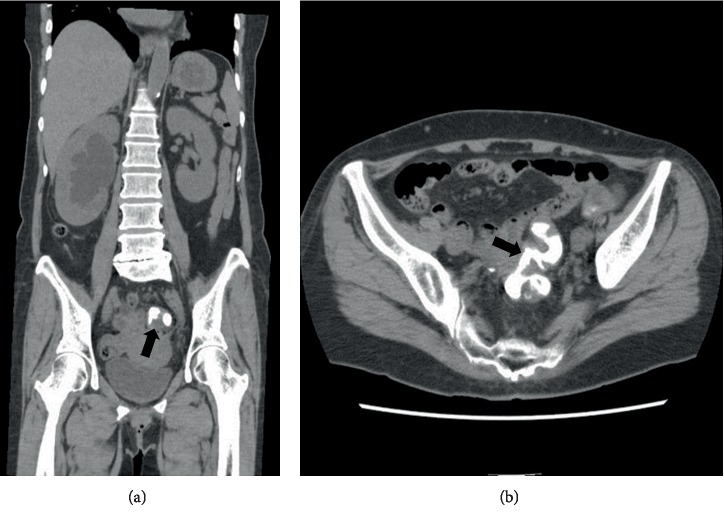
Coronal (a) and axial (b) scan of pelvic CT after retrograde pyelography. Contrast used for retrograde pyelography remains in the sigmoid colon (arrow).

Computed tomography showed remaining of contrast in the sigmoid colon as well as right hydroureteronephrosis, also suggestive of ureterocolic fistula (Figure 4).

**Figure 5:**
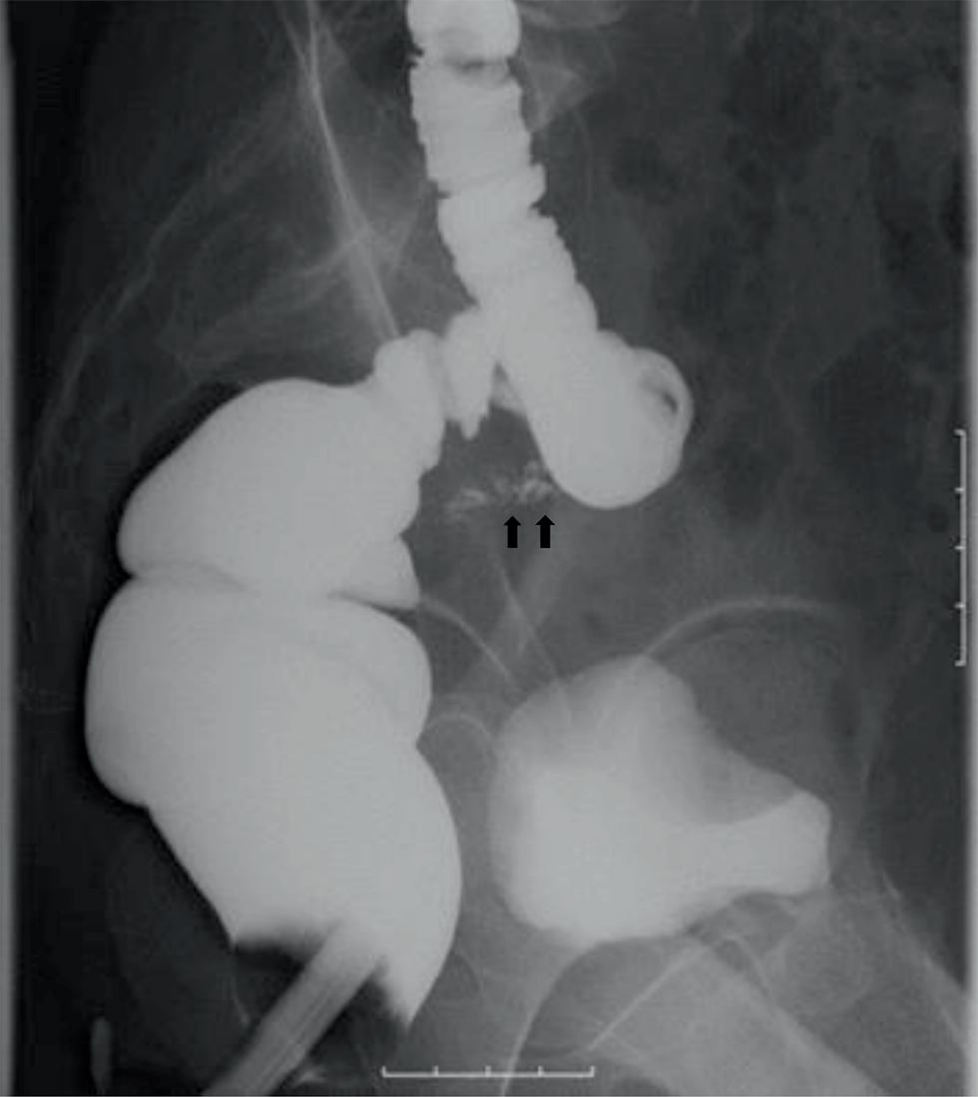
Barium enema showing contrast leakage from the sigmoid colon (arrow).

Barium enema confirmed the contrast leakage from the sigmoid colon (Figure 5).

An attempt at placing a ureteral double J stent was unsuccessful due to severe stricture, and a percutaneous nephrostomy tube was placed in the right kidney, followed by an exploratory laparotomy six days thereafter. Severe adhesion and abscess existed between the cervix and the right pelvic sidewall. Hysterectomy, sigmoid resection, and right ureteroneocystostomy were done. The pathological examination confirmed presence of diverticulitis of the sigmoid colon. The patient was discharged on postoperative day 11, and she has been doing well without any further urinary or intestinal problems.

## 3. Discussion

Iatrogenic ureteral injuries constitute a serious complication of surgery and commonly result from gynecologic procedures. They might be of many varieties such as transection, ligation, crush, resection, and perforation. Ureteral obstruction and fistula formation will usually present later in the postoperative course. According to the review by Ostrzenski et al., less than 10% of ureteral injury cases were diagnosed intraoperatively during the initial laparoscopic surgery whereas more than 70% postoperatively [1]. Thermal injury tends to lead to a delayed clinical appearance [2, 3], which can be explained by the fact that electrocautery instrumentation damages vascular supply beyond the area of actual contact, resulting in delayed necrosis, scarring and partial obstruction of the ureteral wall [2]. This delay could make it unable for immediate intraoperative cystoscopy or early postoperative ultrasonography to detect any injury. Thus, if an injury remains undetected over the postoperative period, it might develop into ureteral obstruction and/or fistula.

Regarding the site of ureteral injury, there is likely to be three locations: (1) at the infundibulopelvic ligament where the ovarian vessels cross the ureter; (2) where the ureter passes deep to the ovarian fossa along the lateral aspect of the uterosacral ligament; and (3) at the ureteral canal passing under the uterine artery [1], the second of which is the case in the present report. For a possible scenario, to explain the development of ureterocolic fistula in the present case, we postulate two steps: first, a ureteroperitoneal fistula was formed, then a ureterocolic fistula developed. As the first step, ureteral damage attributed to adhesiolysis by blunt detaching with occasional bipolar coagulation seems to have caused a ureteroperitoneal fistula. The extravasated urine triggered pseudocyst formation around the fistula, which is likely to correspond to a cystic area detected by ultrasound on postoperative day 34. The pseudocyst grew and then stuck to the edge of the resected right tube, from where urine flowed into the uterine cavity. This process is consistent with the complaint of thin vaginal discharge and the decrease in size of the cyst on postoperative day 62.

The patient had no prior history of diverticulitis. CT scan performed before the first operation (salpingo-oophorectomy) did not detect any diverticulum in the sigmoid colon either. It is hard to measure precise prevalence of colonic diverticulosis because most patients with anatomical diverticulosis remain asymptomatic throughout their lifetime. Assessments by routine screening for colon cancer reported prevalence of 40–60% of people in the US or Europe [4, 5] whereas around 25% in Japan [6]. Of the few who develop complications, diverticulitis is the most usual manifestation, affecting 10–25% of patents with diverticula [7]. Prevalence of diverticular disease increases with age, from less than 10% in people younger than age 40 years to 50–66% in patients older than age 80 years [7].

The next scenario is as to how the above-mentioned ureteroperitoneal fistula developed into a ureterocolic fistula. Once the pseudocyst shrank, it became easier for the sigmoid colon to get close to the ureteroperitoneal fistula. For some reason, adhesion occurred between an unrecognized diverticulum and the adhesiolysed peritoneum around the ureteroperitoneal fistula. The newly formed adhesion triggered diverticulitis, which then developed into perforation of the diverticulum and abscess formation, followed by fistula formation with the adjacent ureter. This process probably developed between postoperative day 62 and 110, during which vaginal discharge gradually decreased while watery diarrhea, appetite loss and right flank pain appeared.

Fistula secondary to diverticulitis occurs in approximately 1% [8]. Colovesical fistula is the most typical with a predominance of men over women, because the uterus effectively shields the diseased colon from the bladder, and most women with colovesical fistula had hysterectomy previously. Compared to colovesical, ureterocolic fistula is extremely rare. Causes of ureterocolic fistula include urinary tract calculi, iatrogenic trauma, radiation therapy, transitional cell carcinoma, tuberculosis, and diverticulitis [9]. A ureteric calculus complicated by obstruction and pyelonephritis is the most common cause. An extensive literature search utilizing PubMed and Ovid databases from 1960 through 2019 revealed that there have been only twelve other published cases of spontaneous ureterocolic fistula secondary to diverticulitis [10–21].  Table 1 summarizes the features of all the twelve cases. In most cases, the left ureter was affected probably due to its proximity to the sigmoid colon, and the present case is the third with the right ureter affected. The present case is unique in that the underlying cause was acute, not chronic, diverticulitis of an unrecognized diverticulum which seemed to be triggered and complicated by an iatrogenic ureteral injury associated with endometriosis.

Regarding iatrogenic ureterocolic fistula following gynecologic surgeries, we were able to find only a single case reported by Floyd et al. [22]. They reported a case of left sided ureterocolic fistula in a 56-year old woman that was diagnosed one month after bilateral laparoscopic oophorectomy. In that case, it is likely that there were no pathological lesions in the ovaries, since the surgery was preventive for a BRCA2-positive patient who had had breast cancer treatment. The presence of colon diverticulum is not mentioned. No detailed information is available about the possible cause of the fistula formation, either. Considering the delayed onset, we assume that, like our case, some ureteral damage probably happened during the procedure using thermal energy devices.

It is preferable to recognize urologic injury intraoperatively; however, this might be difficult and not always possible. Early recognition may decrease subsequent morbidity, which tends to be directly related to the amount of delay. In retrospect, the hypoechoic cystic shadow on the right lateral aspect of the uterus by transvaginal ultrasound on postoperative day 34 (Figure 1) seems to be a sign that ureteral injury happened. If evaluation by such as retrograde pyelography had been performed and ureteral injury had been recognized at that point, ureteral stenting might have prevented urine extravasation and promoted ureteral healing. Alternatively, if abdominal ultrasound or computed tomography had been performed sometime between day 34 and 137, it seems probable that the ureteral damage had been found earlier. In this regard, the present case highlights the need for a high level of suspicion and vigilance which could lead to prompt recognition of urologic injuries. Also, considering increasing incidence of diverticular disease in an aging population, it is important to be aware of a rare presentation like this case.

## Figures and Tables

**Table 1:** Cases of ureterocolic fistula secondary to diverticulitis.

Author/year	Age	Sex	Underlying disease	Laterality of fistula	Management
Rodkey 1965 [10]	NS	NS	NS	NS	NS
Colcock 1972 [11]	72	F	Peritonitis due to diverticulitis	L	Died^∗^
Krishna 1977 [12]	88	F	Perforation due to diverticulitis	L	Spontaneous closure
Heney 1984 [13]	80	M	NS	R	Sigmoid resection, ureter reimplantation
Noordzij 1991 [14]	74	F	Previous radiation therapy due to cervical cancer	L	Nephroureterectomy
Cirocco 1994 [15]	67	F	Chronic diverticulitis	L	Sigmoid resection, ureteral stent
Maeda 1998 [16]	45	F	Renal calculi	L	Sigmoid resection, nephroureterectomy
Osawa 2007 [17]	52	M	Peritonitis due to diverticulitis	L	Sigmoid resection, ureteral stent
Dowling 2009 [18]	79	F	Chronic diverticulitis	L	Sigmoid resection
Lang 2012 [19]	72	M	NS	L	NS
Pai 2014 [20]	80	F	Chronic diverticulitis	L	Sigmoid resection, ureteral stent
Almerie 2015 [21]	68	M	Chronic diverticulitis	R	Sigmoid resection, ureteral stent
Present	62	F	Acute diverticulitis associated with endometriosis	R	Sigmoid resection, ureter reimplantation

NS: not specified. ^∗^The patient died from hemorrhage. The fistula was found at autopsy.
